# The effect of weight controllability beliefs on prejudice and self-efficacy

**DOI:** 10.7717/peerj.1764

**Published:** 2016-03-03

**Authors:** Einar B. Thorsteinsson, Natasha M. Loi, Dana Breadsell

**Affiliations:** Psychology/School of Behavioural, Cognitive and Social Sciences, University of New England, Armidale, Australia

**Keywords:** Exercise, Obesity, Prejudice, Self-efficacy, Weight management

## Abstract

An experiment was conducted to test for the presence of prejudice towards obesity and whether weight controllability beliefs information reduces this prejudice and impacts on a person’s own healthy eating self-efficacy. The experiment randomly allocated 346 participants (49 males) into one of three conditions: controllable contributors toward obesity condition (e.g., information about personal control about diet and exercise); uncontrollable contributors toward obesity condition (e.g., information about genes, factors in society); and a control condition with no information given. Prejudice was present in 81% of the sample. High prejudice was predicted by low self-efficacy for exercise and weight. Weight controllability beliefs information had no significant effect on prejudice levels or exercise or healthy eating self-efficacy levels. Future research directions are discussed.

## Introduction

The World Health Organisation (2015) reports that worldwide obesity rates have nearly doubled since 1980. In 1995, 56.3% of Australian adults were overweight or obese and this rate has increased in 2011–2012 to 62.8%. This figure comprises 35.3% overweight and 27.5% obese adults (Australian Bureau of Statistics, 2013). As rates of obesity have increased, so too have people’s experiences of prejudice (Puhl & Heuer, 2010), with the view that obese individuals are responsible for their obesity (Brownell et al., 2010), and the value Western society places on thinness and health (Gronning, Scambler & Tjora, 2013). The evidence that prejudice directed at obese individuals is present in Western society is generally well documented (Puhl & Heuer, 2009). Research has found that this prejudice can be harmful, potentially reducing a person’s ability to perform the healthy behaviours necessary to lose weight (e.g., Lewis et al., 2011). However, it should be noted when it comes to dieting, it may not lead to better health as such, and given its association with unhealthy behaviours such as disordered eating, it may in fact have negative consequences (e.g., Loth et al., 2014). Thus, dieting behaviour can be used as an indicator for the ‘desire’ to lose weight but not necessarily used to achieve a healthier life.

While research evidence suggests that genetic (physiological) and environmental factors outside the individual’s control can impact a person’s weight (Dubois et al., 2012), there are also perceptions that weight is manageable through healthy eating and being physically active (Swinburn et al., 2004). From a public health perspective, it is important to consider how weight attributions influence peoples’ health. Wang, Brownell & Wadden (2004), for instance, note that unlike other prejudices such as race, obese people often share the same negative stereotypes of obesity as healthy weight individuals. This is further supported by a large-scale study conducted by Schwartz et al. (2006) who reported a pervasive “anti-fat” bias among even their most obese participants. Supporting this are recent findings suggesting that the higher the perception of being overweight the higher the fear of being the victim of prejudice and thus the lower the self-efficacy for being able to control their food intake (Major et al., 2014).

Weiner, Perry & Magnusson (1988) posit that people habitually attribute controllable causality to obese people therefore attributing blame to obese people and prejudicing them for it. These weight controllability attributions are fed by Western societal values of individualism and self-determination, prizing the belief that the individual is responsible for their own life and will get what they deserve (Puhl, Schwartz & Brownell, 2005). The attribution theory of prejudice posited by Weiner, Perry & Magnusson (1988) suggests that when the attribution of controllability is reduced, prejudice towards obesity will be reduced. However, this may create a dilemma where reducing personal control over weight and personal responsibility (Weiner, Perry & Magnusson, 1988) may reduce self-efficacy in healthy weight and exercise management, with self-efficacy being an important predictor of eating behaviour (Glasofer et al., 2013). Research by Dar-Nimrod et al. (2014) suggests that exposing participants to information about genetic causation of weight may increase food consumption. However, it may be worth noting that the participants in this study were undergraduate students and no information about weight status or stigma was provided. If the goal of reducing weight stigma levels is achieved through education about how weight is not completely within a person’s control, it is important to ensure a reduction in healthy eating self-efficacy or exercise is not an unintended consequence. Healthy eating self-efficacy does not measure how healthy an individual’s diet is but rather the individual’s perception of control over eating behaviour.

When overweight participants are primed with weight-related stereotypes their intentions to improve their dietary and exercise-related behaviours are diminished (Seacat & Mickelson, 2009). The underlying factors explaining these effects are not clearly understood (Schmader, Johns & Forbes, 2008). However, being reminded about your ‘shortcomings’ may cause increased levels of stress, increased negative self-assessment, such as thinking about past failures to improve health, and increased unhappiness about current body image (for discussion on some of these points see Schmader, Johns & Forbes, 2008). These findings are backed up by studies where overt weight stigma, such as inappropriate and negative comments from doctors, family, and friends, has been associated with increased rates of binge eating and poor treatment outcomes in the analysis of a 14-week behavioural weight loss program (Wott & Carels, 2010). An Australian study asking obese people what ways prejudice impacted on their lives (Lewis et al., 2011) found that it impacted on emotional health and wellbeing, especially self-worth and self-esteem. Feelings of depression, sadness, anxiety, worry, and loneliness were noted, as well as trouble forming new relationships and less social support.

The impact of weight stigma on exercise motivation and behaviour was studied by Vartanian & Shaprow (2008) who found the more people experienced stigma, the higher their motivation to avoid exercise when controlling for Body Mass Index (BMI) and body dissatisfaction. Vartanian & Novak (2011) found the relationship between experiencing weight stigma and avoiding exercise was moderated by internalisation of the stigma. Internalisation is the individual’s endorsement of societal standards of “attractiveness.” Puhl, Moss-Racusin & Schwartz (2007) found that overweight and obese women who had internalised weight stigma (i.e., believed weight-based stereotypes) were more likely to binge eat and increase their intake of unhealthy food. Thus evidence supports the view that weight stigma does not motivate healthy behaviours but rather suggests that an individual’s confidence to make changes is likely to be effected if they internalise society’s stereotypes regarding overweight and obese.

Danielsdottir, O’Brien & Ciao (2010) found mixed support for interventions designed to reduce prejudice towards obesity, indicating limited support for the notion that reducing blame will alter prejudice levels. In a meta-analysis examining the effectiveness of weight bias interventions, Lee, Ata & Brannick (2014) found that these interventions have a small, but positive, effect on weight attitudes and beliefs. However, these studies tended to be flawed with many methodological issues noted such as the lack of randomised control designs, pre- and post-intervention measures of prejudice not being assessed, and control conditions not utilised. Some success has been reported in successfully changing participants’ genetic causal beliefs but this change was not followed by a change in prejudice towards obesity (Lippa & Sanderson, 2013). Additionally, beliefs that eating habits and lack of exercise contributed toward obesity have been addressed, with higher levels of prejudice supporting the relationship between controllability beliefs and prejudice towards obesity. Swift et al. (2013) reported that following an intervention involving participants watching anti-stigma films, health professionals’ beliefs about weight being under an obese person’s control were reduced and this change was maintained when measured six weeks later. Prejudice towards obesity was also reduced post-intervention but returned to baseline levels within six weeks. The present study attempts to address these methodological concerns.

The present study tries to address methodological flaws of previous studies by employing randomised control trial design including a control condition and by measuring prejudice and other variables before and after intervention. The study design should enable us to test if healthy eating or exercise self-efficacy is lowered as a consequence of reduced weight stigma. No research has been identified that has measured the impact of weight controllability beliefs information on the stigmatiser’s own self-efficacy in performing healthy behaviours. It is important to examine self-efficacy and its potential role in prejudice increasing our theoretical understanding of prejudice and our ability to model and reduce prejudice. If individuals understand that they have control over their own health, in many situations their self-efficacy in relation to health could be expected to improve as well as and concurrently with their motivation and intentions to change their behaviour for the betterment of their health.

### Hypotheses

The following hypotheses were proposed. First, prejudice towards obesity would be present in the sample, establishing the extent of prejudice in relation with previous findings. Second, there would be a relationship between prejudice towards obesity and exercise and healthy eating self-efficacy. Third, using randomised control trial design, a condition emphasising controllable contributors toward obesity would increase the level of prejudice towards obesity and a condition emphasising uncontrollable contributors would reduce the level of prejudice compared to a control condition. Fourth, the condition emphasising controllable contributors toward obesity would increase the level of exercise and healthy eating self-efficacy and the condition emphasising uncontrollable contributors toward obesity would reduce the level of exercise and healthy eating self-efficacy compared to the control condition.

## Method

### Participants

A total of 447 participants (Time 1) were recruited through online notices (university learning management systems), email, and word of mouth. Participation was voluntary and participants were eligible to enter a prize draw to win a $50 iTunes or Kindle voucher. Ages ranged from 18 to 78 years of age (*M* = 36.53, *SD* = 13.27) and there were 72 males and 375 females. Participants’ (Time 1) education levels were high with 17% having a postgraduate degree, 36% with a Bachelor’s degree, 18% with a vocational qualification, 24% with a Higher School Certificate, and 4% with a School Certificate or less.

At post-intervention (Time 2), 346 cases were matched to Time 1 cases. The attrition rate for the current study was 22.6%. The Time 1 only participants (non-completers) were not significantly different (two-tailed tests) from completers. That is, participants who completed both Times 1 and 2 in relation to age (*p* = .828), education (*p* = .501), exercise self-efficacy (*p* = .957), fat phobia (*p* = .951), or weight efficacy lifestyle (*p* = .123). The dataset combining Time 1 and Time 2 consisted of 49 males and 297 females. Male ages ranged from 18 to 67 (*M* = 37.29, *SD* = 13.65) and female ages ranged from 18 to 78 (*M* = 36.68, *SD* = 13.12). The study was approved by the university’s human research ethics committee, HE13-059.

### Materials

Participants were asked to provide their sex, age, and highest level of education achieved. They were then asked “Do you perceive yourself to be of a healthy weight” on a scale of 1 (*Not at all healthy*) to 6 (*Very healthy*). We used this short measure rather than a longer published measure to try and reduce the time commitment of participants increasing the chances of retaining them for Time 2. The mean score of 3.85 (*SD* = 1.52), indicated the average weight perception of participants was rated slightly above moderately healthy. Three scales were utilised at Time 1 and repeated at Time 2.

The *Fat Phobia Scale Short Form* (FPS; Bacon, Scheltema & Robinson, 2001) is a 14-item, 5-point semantic differential scale used to measure attitudes towards obesity. To assess these attitudes toward people with obesity, participants were asked to rate the items (adjective pairs) indicating how the words best described their feelings and beliefs about obese or fat people. Examples include “lazy versus industrious” and “willpower versus no willpower.” FPS scores range from 1 to 5, with 5 representing a high level of prejudice. Bacon, Scheltema & Robinson (2001) reports Cronbach’s alpha of .87 and .91 in their two samples. The FPS in the present study had a Cronbach’s alpha of .92.

Bacon, Scheltema & Robinson (2001) note that a score of 3.60 on the 14-item FPS indicates an average amount of fat phobia. However, subsequent studies have suggested that scores below 2.50 indicate more positive attitudes toward obese people, while scores above 2.50 indicate more negative attitudes (Puhl, Wharton & Heuer, 2009). Thus scores above 2.50 are used in the present study to indicate the presence of weight stigma (i.e., fat phobia).

The *Exercise Self-Efficacy Scale* (ESE; Bandura, 2006, cited and adapted by Everett, Salamonson & Davidson (2009) is an 18-item measure scored on an 11-point Likert scale assessing a person’s exercise self-efficacy by asking participants to rate their level of confidence that they can exercise on a regular basis when given hypothetical situations. For example, “Rate your degree of confidence that you can perform exercise when feeling tired.” Ratings range from 0 (*Cannot do at all*) to 10 (*Certain can do*). Joseph et al. (2014) studied the psychometric properties of the ESE on a sample of undergraduate university students and found a Cronbach’s alpha of .89. The ESE scale in the present study had a Cronbach’s alpha of .95.

The *Weight Efficacy Lifestyle Questionnaire* (WEL; Clark et al., 1991) contains 20 items scored on a 10-point Likert scale measuring a person’s perception of their self-efficacy as concerns eating behaviour. The scale asks participants to rate their level of confidence that they would not eat food in a number of hypothetical situations. Ratings range from 0 (*Not confident*) to 9 (*Very confident*). The WEL has five subscales consisting of: negative emotions (e.g., “I can resist eating when I am anxious or nervous”); availability (e.g., “I can control my eating on the weekends”); social pressure (e.g., “I can resist even when I have to say ‘no’ to others”); physical discomfort (e.g., “I can resist eating when I am in pain”); and positive activities (e.g., “I can resist eating when I am reading”). Cronbach’s alphas for the subscales range from .79 to .88 (Clark et al., 1991). Predictive validity was supported by Andrade et al. (2010), Clark et al. (1991) and Warziski et al. (2008) who found the scale accurately predicted weight loss. In the present study, the total WEL scale was used and had a total Cronbach’s alpha of .94.

### Procedure

**Figure 1:**
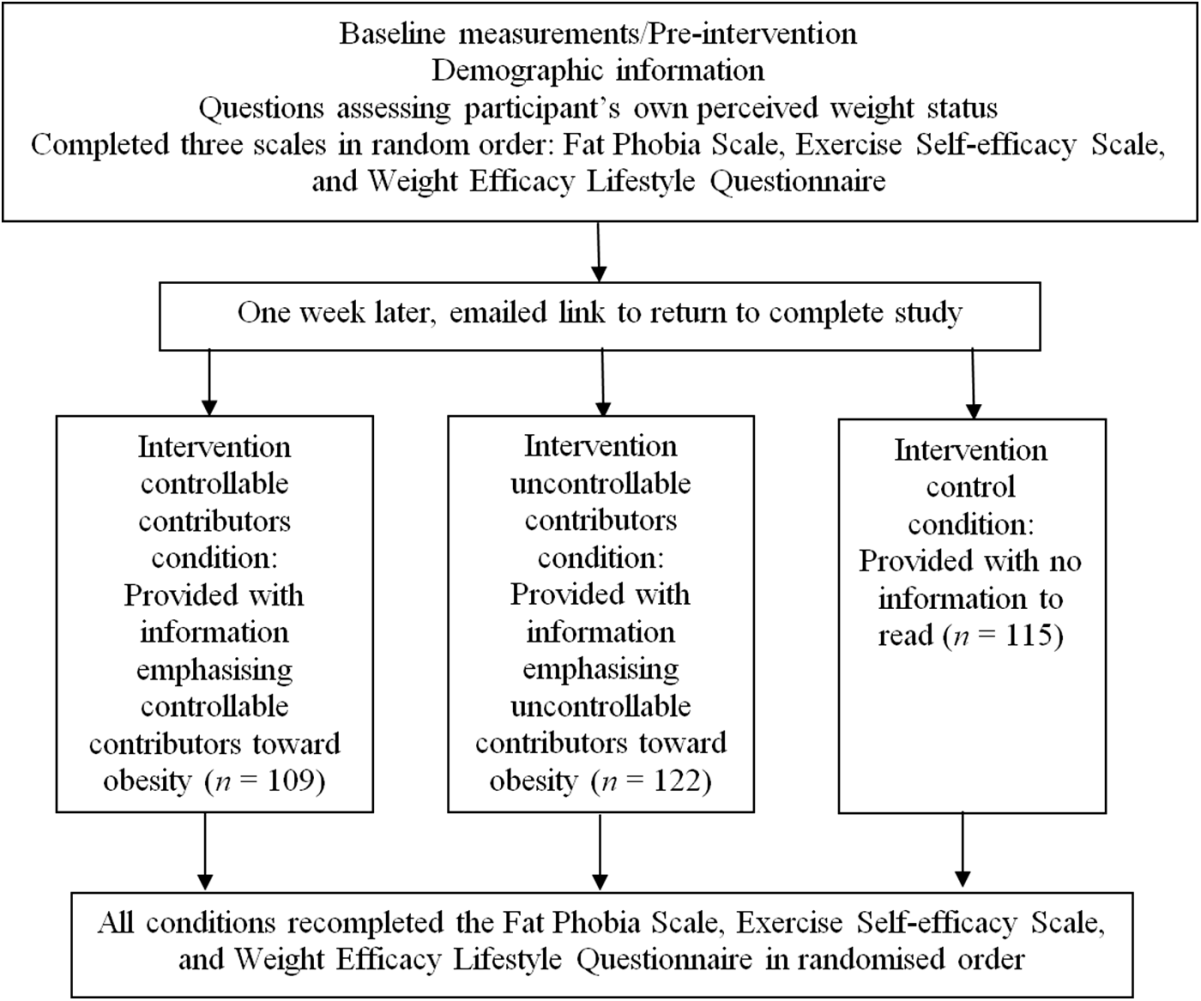
Schematic overview of study design and measurement times. One week later, participants followed the email link to complete the study.

Figure 1 shows a flow chart of the randomised control trial design. Prior to beginning the study, participants were reminded that participation was voluntary and they could withdraw at any time. Clicking on a “Proceed to study” button constituted informed consent. Participants completed the pre-intervention baseline measures at Time 1 and were asked to provide an email address so they could be sent a link to return in a week’s time and complete the second part of the study at Time 2. To ensure anonymity, participants’ email addresses were collected via a conduit, disconnected from any data collected. Times 1 and 2 responses were match using a unique code based on several questions answered by the participants. Typical questions might provide parts of the code such as the first two letters in the town/city you were born in and the last two letters of your mother’s maiden name.

Participants were randomly allocated to one of three conditions: (a) reading a half page essay on uncontrollable factors contributing towards obesity, (b) reading a half page essay on controllable factors contributing towards obesity, and (c) control (not required to read anything). Essay information was sourced from the Australian Government Department of Health (2009) and NSW Department of Health (2013), and partially utilised the weight controllability information by Lippa & Sanderson (2013). In the uncontrollable condition, the information given showed that the environment (e.g., factors in society such as food prices favouring unhealthy food and advertisements), and not the individual, was to blame for weight and that weight was due to genes (e.g., scientific evidence). Meanwhile, in the controllable factors condition, the information given emphasised that weight is under personal control (e.g., diet, exercise) and, as such, a treatable condition. The uncontrollable and controllable material presented was matched in terms of being backed by science, with one suggesting ‘forced’ lifestyle choices and the other suggesting ‘unforced’ lifestyle choices. To make sure that participants had engaged in the material presented, they were asked two questions based on the material corresponding to the intervention as a manipulation check. Two participants failed to answer these two questions and were eliminated from any analysis. At post-intervention, participants completed the same measures, presented in random order, as at Time 1. Participants were fully debriefed at the conclusion of the study and provided with further information on obesity as required.

## Results

Negative attitudes towards obesity were examined at Time 1 and found to be high (*M* = 3.37, *SD* = 0.79) and statistically different from the weight stigma score of 2.50 as suggested by Puhl & Heuer (2009), *t*(446) = 23.18, *p* < .001, with a large effect size Hedges’ *g* = 1.10. There were no statistically significant differences at Time 1 between males (*M* = 3.34, *SD* = 0.79) and females (*M* = 3.37, *SD* = 0.79) for weight stigma, *t*(445) = 0.33, *p* = .744 (two-tailed), Hedges’ *g* = 0.04. This attitude was found to be present in 81% or 362 of the 447 participants, suggesting a high prevalence of negative attitudes toward people with obesity. The same pattern of findings was observed when controlling for sex and were of similar magnitude for Time 2, Hedges’ *g* = 1.32 and prejudice towards obesity present in 90% of participants.

At Time 1, Spearman’s rho indicated that prejudice towards obesity was negatively related to exercise and healthy eating self-efficacy. Table 1 shows that the higher the prejudice, the lower the level of exercise self-efficacy and healthy eating self-efficacy. In addition, the greater the exercise self-efficacy, the greater the healthy eating self-efficacy.

**Table 1:** Summary of Spearman’s rho correlation results at Time 1 (n = 447).

Measure	1	2	3	4
1. Prejudice towards obesity (fat phobia)	–	−.15**	−.20***	−.11*
2. Exercise self-efficacy		–	.47***	.39***
3. Healthy eating self-efficacy			–	.47***
4. Weight perception				–

**Notes:**

The same pattern of findings was observed within males and females. Weight perception = the higher the score the more positive the weight perception.

* *p* < .05.

** *p* < .01.

*** *p* < .001.

Table 1 also showed that, (a) the healthier a person’s rating of their weight perception, the lower their level of prejudice towards obesity, and (b) the healthier a person’s weight perception, the higher they rated their exercise self-efficacy and healthy eating self-efficacy.

To determine whether weight controllability information altered levels of prejudice towards obesity, a one-way ANCOVA compared the post-intervention FPS scores of the three experimental conditions while controlling for pre-intervention scores (see Table 2). The information had no statistically significant effect on prejudice levels at post-intervention, *F*(2,342) = 0.83, *p* = .435, partial η^2^ < .01. Furthermore, there was no significant effect on the level of exercise self-efficacy, *F*(2,342) = 1.04, *p* = .356, partial η^2^ = .01 or healthy eating self-efficacy, *F*(2,342) = 0.04, *p* = .961, partial η^2^ < .01. Examining the information effect within males and females showed a medium effect size, but no statistical significance, in relation to prejudice, *F*(2,45) = 2.36, *p* = .106, partial η^2^ = .10, whereby males in the controllable condition tended to have lower prejudice than their counterparts in the uncontrollable condition, *p*_Sidak_ = .104. Examining sex as a factor did not show any sex by condition interactions for any outcome.

**Table 2:** Summary of the pre-intervention and post-intervention means and standard deviations of Prejudice towards Obesity (PO), Exercise and Healthy Eating Self-Efficacy (ESE), and Weight Efficacy Lifestyle (WEL) levels by conditions (n = 346).

	Pre-intervention (*M, SD*)			Post-intervention (*M, SD*)		
	Controllable	Uncontrollable	Control	Controllable	Uncontrollable	Control
PO	3.34 (0.84)	3.39 (0.78)	3.38 (0.78)	3.44 (0.81)	3.56 (0.72)	3.48 (0.73)
ESE	91.86 (40.82)	84.49 (37.26)	90.30 (38.80)	94.21 (40.08)	85.02 (37.21)	89.43 (36.90)
WEL	6.08 (1.67)	5.81 (1.67)	5.91 (1.63)	6.03 (1.73)	5.76 (1.65)	5.87 (1.55)

**Note:**

Controllable information (*n* = 109); Uncontrollable information (*n* = 122); Control condition (*n* = 115).

Post hoc analyses were also conducted exploring age, sex, and education. The younger the participant, the more healthy they rated their weight perception, *r_s_*(445) = −.10, *p* = .032 (two-tailed), and the older the participant the higher their level of prejudice towards obesity, *r_s_*(445) = .14, *p* = .003 (two-tailed). Also, the more educated the participant, the higher their level of prejudice, *r_s_*(445) = .11, *p* = .025 (two-tailed). The sex of participants was found to be unrelated to either weight perception, level of prejudice towards obesity, exercise, or healthy eating self-efficacy.

## Discussion

Based on the weight stigma score suggested by Puhl & Heuer (2009), the current study confirms the pervasiveness of prejudice towards obesity in a well-educated sample. The high prevalence of prejudice, with 81% of the sample scoring higher than 2.50 at Time 1, combined with the fact that research has found it is harmful to people’s health (Sutin & Terraciano, 2013; Wott & Carels, 2010), confirms the importance of researching interventions aimed at reducing its occurrence.

The present study hypothesised that participants’ levels of prejudice toward obese people would be related to their own exercise and healthy eating self-efficacy levels. This relationship has not previously been investigated and is, therefore, exploratory in nature. This hypothesis was supported with a significant negative relationship found between prejudice, exercise, and healthy eating self-efficacy. Thus the higher the participant’s level of prejudice, the lower their levels of exercise and healthy eating self-efficacy. High self-efficacy (i.e., exercise and weight) may indicate underlying happiness and wellbeing that in turn promotes increased tolerance and less prejudice towards individuals that are different from you. Furthermore, if you are happy about your own situation (e.g., weight) you may feel sorry for those that are less fortunate than you. A small but significant negative relationship between participants’ weight perception and their levels of prejudice towards obesity was found. Thus the healthier a person rated their weight, the lower their level of prejudice. This is an interesting finding given that healthy weight individuals should be more likely to consider weight as controllable and therefore be unsympathetic to those unable to successfully control their own weight. However, this was not demonstrated in the current study. This finding should be interpreted with caution, though, given that the relationship was small (*r* = −.11) and the potential concerns relating to the accuracy of reported weight (especially in women) found in some research (e.g., Engstrom et al., 2003).

Additionally, a significant positive relationship between participants’ weight perception and levels of exercise and healthy eating self-efficacy was found indicating that the healthier a person rated their own weight, the higher their own level of exercise and healthy eating self-efficacy. This was consistent with a strong positive relationship between exercise and healthy eating self-efficacy. These relationships suggest that there may be a ‘general’ efficacy factor explaining the correlation between exercise and weight efficacy.

The current study also predicted that information emphasising the *controllable contributors* toward obesity (i.e., explaining how obese people are to blame) would increase prejudice, while information emphasising the *uncontrollable contributors* toward obesity (i.e., explaining how obese people are not to blame) would reduce prejudice. Weight controllability beliefs information had no effect on levels of prejudice in either the controllable or uncontrollable conditions when compared to a control. Therefore, this hypothesis was not supported.

The absence of change in prejudice levels in response to weight controllability beliefs information indicates that attribution theory alone does not explain or alter prejudice towards obesity. Weight controllability attributions could play a role in the development of prejudice towards obesity. However, this relationship is complex and mediated by other variables yet to be fully understood. This is demonstrated through studies finding that weight controllability beliefs information reduces negative trait ratings but fails to improve positive trait ratings (Puhl, Schwartz & Brownell, 2005), that changes in causal beliefs resulted in no changes in prejudice levels (Lippa & Sanderson, 2013), and that while reduced causal beliefs about obesity and improved prejudice levels occur post-intervention, these levels return to baseline levels mere weeks later (Swift et al., 2013). Several different techniques for altering controllability beliefs have been attempted including lectures, weekly tutorials, and videos, but no weight controllability beliefs intervention has yet consistently demonstrated altered levels of prejudice towards obesity (Danielsdottir, O’Brien & Ciao, 2010). However, the focus needs to be on the message delivered. A recent study reported that if the overweight person was seen to be putting in the effort they might be subjected to less prejudice (Black, Sokol & Vartanian, 2014). Finally, the finding that weight controllability information did not alter people’s levels of exercise or healthy eating self-efficacy for the worse is reassuring to any potential incorporation of weight controllability beliefs information into future interventions. The lack of support for this hypothesis should not necessarily mean that it is not a potentially useful method for reducing prejudice towards obese people. Future research may merely need to consider a manipulation that more emphatically emphasises weight controllability beliefs information.

The present study also tested whether weight controllability beliefs information used in interventions designed to reduce prejudice towards obesity would influence participants’ levels of self-efficacy in managing their own weight. The hypothesis that information emphasising controllable contributors toward obesity would increase exercise and healthy eating self-efficacy and information emphasising uncontrollable contributors toward obesity would reduce exercise and healthy eating self-efficacy when compared to a control condition was not supported.

Weiner, Perry & Magnusson (1988) noted that educating people about the uncontrollable determinants of obesity could create a dilemma. When interventions attempt to reduce personal responsibility for obesity, they do not promote personal change, nor do they support self-efficacy in performing healthy behaviours. Therefore, the impact of interventions on people’s ability to manage their own weight should be vigilantly monitored in future. Even if personal responsibility for obesity status is reduced, this should not be considered incongruent with personal responsibility for healthier lifestyle choices such as an active life (e.g., walking, gardening, not sitting at work). This is supported by findings suggesting that lack of personal responsibility for weight could potentially increase food consumption (Dar-Nimrod et al., 2014).

Finally, in line with the arguments presented by Haidt (2001), prejudice may simply be the judgement given by participants unaffected by any education (i.e., the intervention in the present study) about factors related to obesity. At the same time, researchers may try to use obesity education to understand prejudice while failing to see that education may have very limited effects on prejudice, if any.

The level of prejudice seemed to increase from Times 1–2 by nine percentage points. A comparison of participants’ demographics between Times 1 and 2 does not suggest that the two samples are very different though it should be noted that the attrition for males was 31.9% while it was 20.8% for females. Alternatively, it is possible that participants who felt more strongly about the issues explored in the present study were more likely to complete both time periods. Furthermore, participants who feel strongly about the issue may also have been less affected by the manipulation. However, given the overall lack of impact by the manipulation it is unlikely that it affected this outcome. The post hoc analyses suggested that the lower the prejudice towards obesity the (a) younger the participants, (b) better the weight perception, and (c) lower the education. These are not exactly robust findings as each explained less than 2% of the variance in prejudice and do not seem consistent with the literature or perceptions in society (e.g., Latner, Stunkard & Wilson, 2005). Variations in the relationship to prejudice across studies might reflect different samples and assessment procedures but these relationships need to be examined further, potentially through a systematic review that can tease out such differences.

### Limitations and future recommendations

The present study contained a high proportion of well-educated female participants suggesting that its findings should not be generalised to the Australian population as a whole. Future research could benefit from investigating healthy weight and unhealthy weight participants of both sexes to further explore how self-efficacy and weight perception are related to prejudice towards obesity. Research also needs to explore how coping with this kind of prejudice could be related to the perpetuation of more prejudice. BMI was not assessed due to the unreliability of self-reported weight and height (e.g., Rothman, 2008). As an alternative, we assessed weight perception given that it is potentially an important factor in prejudice (Major et al., 2014). However, measuring BMI and/or some type of waist to height ratio measure would be beneficial in future studies. The control condition did not control for reading a short ‘essay’ unrelated to weight issues but it did allow control for time passed and effects of reading the different questionnaire items. This suggests that future studies should consider employing a similar but unrelated manipulation in the control condition. Future research in this area also needs to seriously consider new methods to reduce prejudice given the mixed findings in the literature (e.g., Aboud et al., 2012).

## Conclusion

Western culture is currently facing a dilemma whereby our cultural value for thinness is pitted against rising obesity rates. Prejudicing obese individuals does not support healthy behaviour and exacerbates their health issues (Drury & Louis, 2002; Vartanian & Novak, 2011; Wott & Carels, 2010). Research is urgently needed to tackle the ubiquitous, publicly acceptable, and ultimately harmful practice of prejudicing people who are overweight or obese.

## Additional Information and Declarations

### Data Deposition

The following information was supplied regarding data availability: Figshare: 1. Thorsteinsson, E. B., Breadsell, D. & Loi, N. M. (2015). Weight controllability effects on prejudice and self-efficacy (Times 1 and 2) [DATA]. figshare. Available at: http://dx.doi.org/10.6084/m9.figshare.1591493. 2. Thorsteinsson, E. B., Breadsell, D. & Loi, N. M. (2015). Weight controllability effects on prejudice and self-efficacy (Time 1) [DATA]. figshare. Available at: http://dx.doi.org/10.6084/m9.figshare.1591492.
